# The effectiveness of the COVID-19 vaccines in the prevention of post-COVID conditions in children and adolescents: a systematic literature review and meta-analysis

**DOI:** 10.1017/ash.2024.42

**Published:** 2024-04-19

**Authors:** Maria Celidonio Gutfreund, Takaaki Kobayashi, Gustavo Yano Callado, Isabele Pardo, Mariana Kim Hsieh, Vivian Lin, Eli N. Perencevich, Jorge L. Salinas, Michael B. Edmond, Eneida Mendonça, Luiz Vicente Rizzo, Alexandre R. Marra

**Affiliations:** 1 Faculdade Israelita de Ciências da Saúde Albert Einstein, Hospital Israelita Albert Einstein, São Paulo, SP, Brazil; 2 Department of Internal Medicine, University of Iowa Carver College of Medicine, Iowa City, IA, USA; 3 Center for Access & Delivery Research & Evaluation (CADRE), Iowa City Veterans Affairs Health Care System, Iowa City, IA, USA; 4 Division of Infectious Diseases & Geographic Medicine, Stanford University, Stanford, CA, USA; 5 Department of Medicine, West Virginia University School of Medicine, Morgantown, WV, USA; 6 Department of Biomedical Informatics, University of Cincinnati, Cincinnati, OH, USA; 7 Division of Biomedical Informatics, Cincinnati Children’s Hospital Medical Center, Cincinnati, OH, USA

## Abstract

**Objective:**

We performed a systematic literature review and meta-analysis on the effectiveness of coronavirus disease 2019 (COVID-19) vaccination against post-COVID conditions (long COVID) in the pediatric population.

**Design:**

Systematic literature review/meta-analysis.

**Methods:**

We searched PubMed, Cumulative Index to Nursing and Allied Health Literature (CINAHL), EMBASE, Cochrane Central Register of Controlled Trials, Scopus, and Web of Science from December 1, 2019, to August 14, 2023, for studies evaluating the COVID-19 vaccine effectiveness against post-COVID conditions among vaccinated individuals < 21 years old who received at least 1 dose of COVID-19 vaccine. A post-COVID condition was defined as any symptom that was present 4 or more weeks after COVID-19 infection. We calculated the pooled diagnostic odds ratio (DOR) (95% CI) for post-COVID conditions between vaccinated and unvaccinated individuals.

**Results:**

Eight studies with 23,995 individuals evaluated the effect of vaccination on post-COVID conditions, of which 5 observational studies were included in the meta-analysis. The prevalence of children who did not receive COVID-19 vaccines ranged from 65% to 97%. The pooled prevalence of post-COVID conditions was 21.3% among those unvaccinated and 20.3% among those vaccinated at least once. The pooled DOR for post-COVID conditions among individuals vaccinated with at least 1 dose and those vaccinated with 2 doses were 1.07 (95% CI, 0.77–1.49) and 0.82 (95% CI, 0.63–1.08), respectively.

**Conclusions:**

A significant proportion of children and adolescents were unvaccinated, and the prevalence of post-COVID conditions was higher than reported in adults. While vaccination did not appear protective, conclusions were limited by the lack of randomized trials and selection bias inherent in observational studies.

## Background

The ongoing global battle against the coronavirus disease 2019 (COVID-19) pandemic has seen remarkable progress in vaccine development and distribution.^1,2^ As adults and high-risk populations receive their COVID-19 vaccinations, the focus has increasingly turned toward vaccinating children and adolescents. While much attention has rightfully been placed on preventing COVID-19 infections in these age groups, it is equally critical to consider the potential impact of vaccination on preventing post-COVID conditions, often referred to as long COVID.

Post-COVID conditions encompass a wide spectrum of persistent health problems that can afflict individuals for weeks, months, or even longer after the initial COVID-19 infection.^3^ According to a published systematic literature review, the prevalence of post-COVID conditions in the pediatric population varies remarkably from 1.6% to 70%.^4^ Long COVID can negatively affect daily function and school attendance.^3,4^

Research has demonstrated the efficacy, safety, and tolerability of COVID-19 vaccines in the pediatric population.^5^ Beyond preventing infection, COVID-19 vaccines also can prevent severe outcomes, including emergency department or urgent care visits, hospitalizations, and even death.^6,7^ Furthermore, vaccination can allow more children to attend school.^5^

As COVID-19 vaccine campaigns have progressed, more children are getting vaccinated. Vaccine effectiveness (VE) assesses the level of protection provided by COVID-19 vaccines against specific conditions.^2^ While VE against post-COVID conditions among the adult population is estimated to be 30%,^8^ VE against post-COVID conditions among the pediatric population is still unknown. Therefore, our objective was to conduct a systematic literature review on the effectiveness of COVID-19 vaccines to prevent post-COVID conditions in children and adolescents, and we pooled the results of published studies to allow for more precise effectiveness estimates.

## Methods

### Systematic literature review and inclusion and exclusion criteria

This review was conducted according to the Preferred Reporting Items for Systematic Reviews and Meta-Analysis (PRISMA) statement^9^ and the Meta-analysis of Observational Studies in Epidemiology (MOOSE) guidelines^10^ and was registered on Prospero (https://www.crd.york.ac.uk/PROSPERO/) on June 9, 2023 (registration number CRD42023456888). Institutional Review Board approval was not required. Inclusion criteria for studies in this systematic review were as follows: original research manuscripts, published in peer-reviewed scientific journals, involved vaccinated (at least 1 dose of COVID-19 vaccines [mRNA or vectorial or inactivated viral vaccine]) and unvaccinated individuals, evaluated the long-term effectiveness of the COVID-19 vaccine, evaluated the pediatric population (individuals < 21 years old), and observational study design. Post-COVID condition (also known as long COVID) was defined as a wide range of health symptoms that are present 4 or more weeks after COVID-19 infection.^3^ The literature search included studies from December 1, 2019, to August 14, 2023. Editorials, commentaries, reviews, study protocols, and studies in the adult population were excluded. Studies without a comparison between vaccinated and unvaccinated individuals (or other vaccinated control groups) were also excluded.

### Search strategy

**Figure 1.**
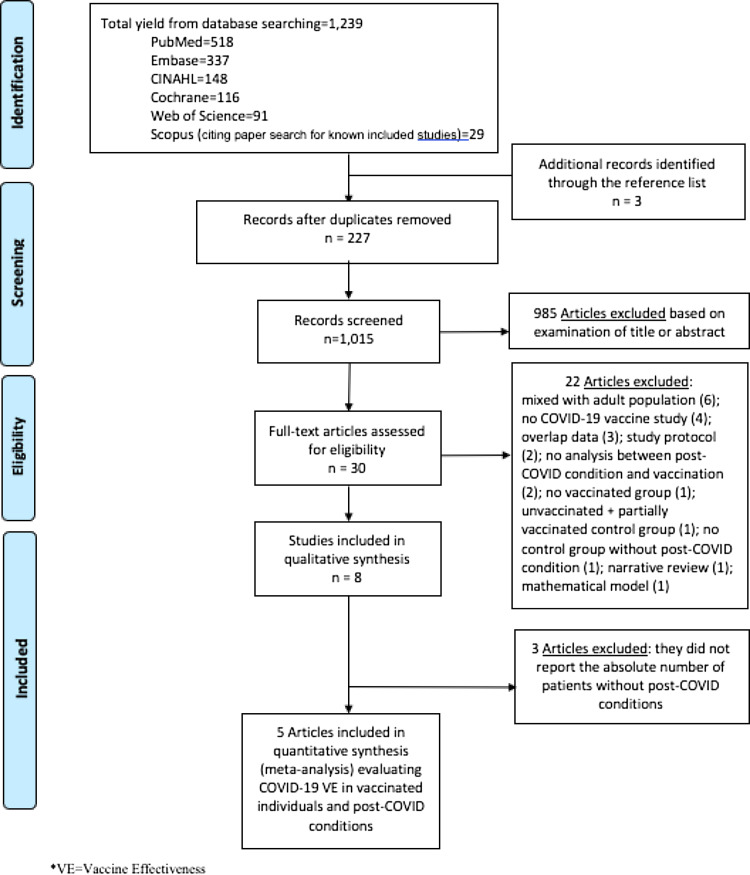
Literature search for articles on the COVID-19 vaccine effectiveness in post-COVID conditions.

We performed literature searches in PubMed, Cumulative Index to Nursing and Allied Health Literature (CINAHL), Embase (Elsevier Platform), Cochrane Central Register of Controlled Trials, Scopus (which includes EMBASE abstracts), and Web of Science. The entire search strategy is described in Supplementary Appendix 1. After applying the exclusion criteria, we reviewed 30 articles, of which 8 met the inclusion criteria and were included in the systematic literature review (Figure 1).

### Data abstraction and quality assessment

Titles and abstracts of all articles were screened to assess whether they met the inclusion criteria. Abstract screening was performed by 2 reviewers (MCG and ARM). Of 7 independent reviewers (ARM, GYC, IP, MCG, MKH, TK, and VL), 2 independently abstracted data for each article using a standardized abstraction form. Reviewers resolved disagreements by consensus.

The reviewers abstracted data on study design, population and location, study period (months) and calendar time, demographic and characteristics of participants, and the types of COVID-19 vaccine administered if available. Post-COVID conditions were considered the primary outcome to calculate VE after at least 1 dose of a COVID-19 vaccine. Eight corresponding authors were contacted for additional information, and 2 were able to provide additional information regarding the number of individuals with and without post-COVID conditions in both vaccinated and unvaccinated groups.^11,12^ The risk of bias was assessed using the Downs and Black scale.^13^ Reviewers answered all original questions from this scale except for question #27 (a single item on the Power subscale scored 0 to 5), which was changed to a yes or no. Two authors performed component quality analysis independently, reviewed all inconsistent assessments, and resolved disagreements by consensus.^14^

### Statistical analysis

To perform a meta-analysis on the extracted data, we calculated the pooled diagnostic odds ratio (DOR) for post-COVID conditions between vaccinated (received at least 1 dose of a COVID-19 vaccine) and unvaccinated individuals. We performed stratified analyses by the timing of the COVID-19 vaccine (ie, those with COVID-19 vaccines before or after COVID-19 diagnosis and those with COVID-19 vaccines before COVID-19 diagnosis) and between those vaccinated with 2 doses and unvaccinated individuals. We performed statistical analysis using R version 4.1.0 with mada package version 0.5.8.^15^ Analogous to the meta-analysis of the odds ratio methods for the DOR, an estimator of random-effects model following the approach of DerSimonian and Laird is provided by the mada package.^15^ For our meta-analysis, we used a bivariate random-effects model, adopting a similar concept of performing the diagnostic accuracy. This enabled simultaneous pooling of sensitivity and specificity with mixed-effect linear modeling while allowing for the trade-off between them.^16,17^ Heterogeneity between studies was evaluated with I^2^ estimation and the Cochran Q statistic test. Publication bias was assessed using the Egger test with R version 4.1.0 with metafor package.^18^

## Results

### Characteristics of included studies

Eight studies met the inclusion criteria^11,12,19–24^ and were included in the final review (Table 1). All studies were non-randomized;^11,12,19–24^ of these, 5 were prospective cohort studies,^12,21–24^ 2 were cross-sectional studies,^19,20^ and 1 was a retrospective cohort study.^11^ Four of these studies evaluated the Pfizer/BioNTech vaccine.^12,21,22,24^ Two analyzed the Moderna vaccine,^21,24^ 1 analyzed the AstraZeneca vaccine,^12^ and 1 analyzed the Sinopharm vaccine.^12^ Four studies did not report the type of COVID-19 vaccine administered.^11,19,20,23^ There were no published studies that evaluated post-COVID conditions as an outcome of bivalent COVID-19 vaccines.

**Table 1.** Summary of characteristics of studies included in the systematic literature review

First author, year, location, study design, study period in # of months and (dates)	COVID-19 vaccine, COVID-19 vaccine before COVID-19 infection	Participants (n) and characteristics	Post-COVID condition		Post-COVID condition		Post-COVID condition definition	Symptoms included in post-COVID condition studies	Benefit of COVID-19 vaccines to decrease post-COVID condition symptoms	D&B score (max = 28)
			Vaccinated at least 1 dose	Control group (unvaccinated)	Vaccinated 2^nd^ dose	Control group (unvaccinated)				
Adler, 2022, Israel Cross-sectional study 2 (Dec 2021–Jan 2022)	NR NR	3,240 participants (1,148 children aged 5–18 years old had a history and 2,092 had no history of COVID-19 infection. Of all the children with a history of COVID-19 infection, 720 [62.7%] had symptomatic COVID-19, 4 were hospitalized due to COVID-19 [0.3%], and 1 needed oxygen supply)	NR	NR	NR	NR	Long duration of COVID-19 symptoms ≥ 4 weeks	-General: fatigue; -Respiratory and heart: difficulty breathing, chest pain, cough, fast beating; -Neurological: difficulty thinking or concentrating (“brain fog”), headache, change in smell or taste, dizziness, sleep problems, mood changes; -Digestive: abdominal pain; -Other: joint or muscle pain, rash	No. COVID-19 vaccination did not reduce post-COVID condition symptoms	24
Atchison, 2023, UK Cross-sectional study 24 (Mar 2020–Mar 2022)	NR Yes	10,059 Participants aged 5–17 years old who reported 1 or more symptoms at onset of COVID-19 (1,051 children and adolescents were symptomatic at 3 months). 97% of the individuals were unvaccinated	339 (42 with long COVID)	9,720 (1,009 with long COVID)	29 (5 with long COVID)	9,720 (1,009 with long COVID)	Long duration of COVID-19 symptoms ≥ 12 weeks	-General: fatigue; -Respiratory and heart: shortness of breath, chest pain, fast beating or pounding heart, cough; -Neurological: difficulty thinking or concentrating (“brain fog”), headache, change in smell or taste, dizziness or lightheadedness, pins-and-needles feelings, sleep problems; -Digestive: abdominal pain, diarrhea; -Other: joint or muscle pain, fever	No. COVID-19 vaccination did not reduce post-COVID condition symptoms	20
Ertesvag, 2023, Norway Prospective cohort study 9 (Aug 2021–Apr 2022)	Pfizer/BioNTech and Moderna Yes	276 participants (All children and adolescents [aged 10–20 years old] had mild, self-limiting delta COVID-19 infection, not requiring hospitalization. At 3 months post-Delta infection, of a subgroup of 89 participants, 56% reported persisting symptoms at 3 months)	NR	NR	NR	NR	At least 1 persisting symptom with a minimum 3-month duration, which impacts everyday functions	-General: fatigue; -Respiratory: dyspnea, cough, chest pain, palpitations; -Neurological: memory and concentration difficulties, headache, change in smell or taste, dizziness, pins-and-needles feeling, sleep problems; -Digestive: diarrhea; -Other: joint or muscle pain, fever	No. COVID-19 vaccination did not reduce post-COVID condition symptoms	23
Jarupan, 2023, Thailand Prospective cohort study 12 (Jul 2021–Jun 2022)	Pfizer/BioNTech No	154 hospitalized participants, being 97 children (5–11 years old) and 57 adolescents (12–17 years old), who reported 25% and 32% of post-COVID condition symptoms, respectively	NR	NR	NR	NR	Long duration of COVID-19 symptoms ≥ 12 weeks	-General: fatigue, post-exertional malaise, fever; -Respiratory and heart: shortness of breath, cough, chest pain, fast beating or pounding heart; -Neurological: difficult thinking or concentrating (“brain fog”), headache, mood changes, change in smell or taste, dizziness or lightheadedness; -Digestive: diarrhea; -Other: joint or muscle pain, rash, changes in menstrual period cycles	NR	18
Lokanuwatsatien, 2023, Thailand Prospective cohort study 6 (Sep 2021–Mar 2022)	Pfizer/BioNTech, AstraZeneca, and Sinopharm Yes	802 Participants (179 [22.3%] were aged 0–3 years and 623 [77.7%] were in the 3–18 age group. The overall prevalence of long COVID was 30.2%). 82.5% of the individuals were unvaccinated	40 (9 with long COVID)	662 (205 with long COVID)	100 (28 with long COVID)	662 (205 with long COVID)	Long duration of COVID-19 symptoms ≥ 12 weeks	-General: fatigue; -Respiratory and heart: difficulty breathing, cough, chest pain, palpitation; -Neurological: headache, change in smell or taste, dizziness, mood changes, pins-and-needles feeling; -Digestive: abdominal pain, diarrhea; -Other: joint or muscle pain, rash	No. COVID-19 vaccination did not reduce post-COVID condition symptoms	23
Messiah, 2022, USA Prospective cohort study 20 (Oct 2020–May 2022)	NR NR	1,828 non-hospitalized participants at 5–19 years old (Texas Coronavirus Antibody REsponse Survey [CARES]). 27 (1.5%) and 58 (3.2%) individuals reported post-COVID condition symptoms of 4–12 weeks and ≥ 12 weeks, respectively. 65% of the individuals were unvaccinated	142 (1 with long COVID)*	1,085 (21 with long COVID)*	614 (11 with long COVID)**	1,110 (46 with long COVID)**	Long duration of COVID-19 symptoms 4–12 weeks*, or ≥ 12 weeks**	-General: fatigue, post-exertional malaise; -Respiratory and heart: shortness of breath, chest pain, cough, fast beating or pounding heart; -Neurological: difficult thinking or concentrating (“brain fog”), headache, change in smell or taste, sleep problem, mood changes; -Other: fever, rash	Yes. COVID-19 vaccination did reduce post-COVID condition symptoms	21
Morello, 2023, Italy Prospective cohort study 34 (Feb 2020–Oct 2022)	Pfizer/BioNTech, and Moderna Yes	1,234 hospitalized or non-hospitalized participants, 0–18 years old at 3, 6, 12, and 18 months of follow-up, respectively. 294 individuals (23.8%) reported post-COVID condition symptoms. 78.8% of the individuals were unvaccinated	261 (81 with long COVID)	973 (213 with long COVID)	261 (54 with long COVID)	973 (240 with long COVID)	Long duration of COVID-19 symptoms ≥ 12 weeks	-General: fatigue; -Respiratory and heart: shortness of breath, cough; -Neurological: difficult thinking or concentrating (“brain fog”), change in smell or taste; -Digestive: abdominal pain, diarrhea; -Other: joint or muscle pain	No. COVID-19 vaccination did reduce post-COVID condition symptoms	21
Pinto Pereira, 2023, UK Retrospective cohort study 6 (Sep 2020–Mar 2021)	NR NR	Of 6,402 non-hospitalized participants aged 11–17 years old with a COVID-19 PCR test positive (UK CLoCk Study Survey) who received COVID-19 vaccine, 576 (9.0%) and unvaccinated 5,826 (91.0%). Post-COVID condition symptoms were reported by 39.6% and 41.9% of vaccinated (at least 1 dose) and unvaccinated individuals, respectively	576 (228 with long COVID)	5,829 (2,443 with long COVID)	NR	NR	The presence of symptoms within 6 months after COVID-19 infection	-General: fatigue; -Respiratory and heart: shortness of breath, cough; -Neurological: headache, change in smell or taste, dizziness, tinnitus; -Digestive: abdominal pain, diarrhea; -Other: joint or muscle pain, rash	No. COVID-19 vaccination did reduce post-COVID condition symptoms	17

Note. COVID-19, coronavirus disease 2019; D&B score, Downs and Black score; NR, not reported; PCR, polymerase chain reaction.

* Long duration of COVID-19 symptoms 4-12 weeks.

** Long duration of COVID-19 symptoms ≥12 week.

Two studies included in our review were conducted in the United Kingdom,^11,20^ and 2 were conducted in Thailand.^12,22^ One study each was performed in Israel,^19^ Italy,^24^ Norway,^21^ and the United States.^23^ All studies were performed between February 2020 and October 2022. The study duration varied from 2 months to 34 months.^11,12,19–24^

In our qualitative analysis, 8 studies including 23,995 children and adolescents evaluated the effect of vaccination among vaccinated and unvaccinated pediatric individuals on post-COVID conditions.^11,12,19–24^ Four studies evaluated VE in pediatric individuals vaccinated only before COVID-19 infection,^12,20,21,24^ 1 study evaluated VE for post-COVID conditions among those who were vaccinated after COVID-19 infection,^22^ and 3 studies evaluated VE but did not specify the timing of the vaccine.^11,19,23^ All 8 studies evaluated VE with at least 1 dose of a COVID-19 vaccine.^11,12,19–24^ Two studies evaluated vaccinated children and adolescents with 2 doses of vaccine.^12,24^ While 5 of 8 studies reported data during the Omicron variant era,^12,20–22,24^ 3 studies took place before the Omicron variant era.^11,19,23^

Each study adopted different definitions for post-COVID conditions (Table 1). Post-COVID conditions were defined as symptoms lasting more than 4 weeks in 2 studies,^19,23^ more than 12 weeks in 5 studies,^12,20–22,24^ and more than 6 months in 1 study.^11^ All studies used at least one of the common post-COVID condition symptoms (details shown in Table 1) to make a diagnosis of a post-COVID condition. Six of the included studies did not show any benefit of COVID-19 vaccination in reducing post-COVID condition symptoms in children and adolescents.^11,12,19–21,24^ One study showed that vaccination was protective against post-COVID-19 symptoms,^23^ and 1 study did not report the benefit of COVID-19 vaccination.^22^

**Figure 2.**
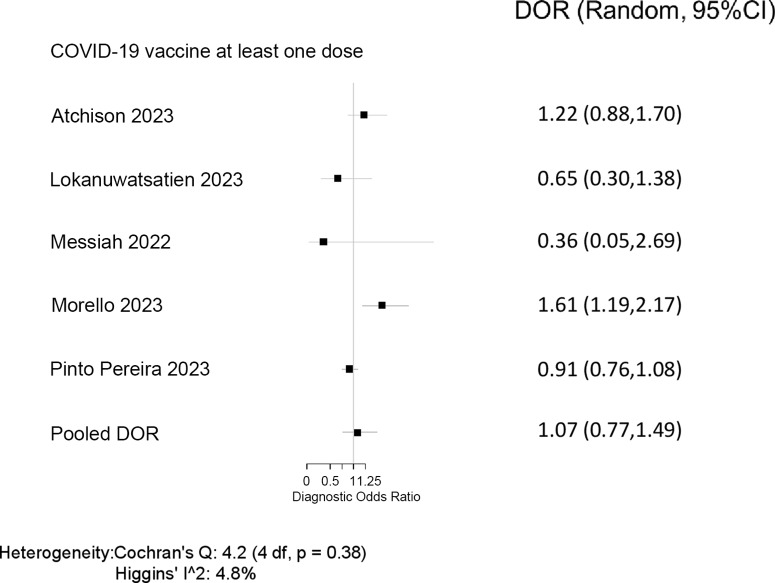
Forest plot of COVID-19 vaccine effectiveness among post-COVID-19 conditions in individuals who received at least 1 dose of COVID-19 vaccine before or after COVID-19 infection. Diagnostic odds ratios (DOR) were determined with the DerSimonian and Laird random-effects method. *Note*: CI, confidence interval.

In total, 5 studies, comprising 20,325 children and adolescents, investigated post-COVID conditions among individuals who had received at least 1 dose of the COVID-19 vaccine either before or after COVID-19 infection. These studies were subsequently included in the meta-analysis (Figure 2).^11,12,20,23,24^ The prevalence of children and adolescents who did not receive COVID-19 vaccines ranges from 65% to 97%.^11,12,20,23,24^ The pooled prevalence of post-COVID conditions was 21.3% among those who were unvaccinated and 20.3% among those who received at least 1 dose.^11,12,20,23,24^ The pooled DOR for post-COVID-19 conditions among the pediatric population vaccinated with at least 1 dose was 1.07 (95% CI, 0.77–1.49).^11,12,20,23,24^ Of the 5 studies, 3 evaluated post-COVID conditions in individuals who received the COVID-19 vaccine before infection (Supplementary Appendix 2).^12,20,24^ The DOR was 1.23 (95% CI, 0.84–1.80).^11,19,23^ Two studies assessed post-COVID conditions for those who received 2 doses before infection (Supplementary Appendix 3).^12,24^ The DOR was 0.82 (95% CI, 0.63–1.08)^12,24^ (Table 2). Because there were no studies evaluating post-COVID conditions for those who received at least 1 dose of each specific type of COVID-19 vaccine (mRNA or viral vector or inactivated viral vaccine), we did not perform a stratified analysis. The results of meta-analyses were homogeneous for studies evaluating post-COVID conditions in individuals who received the COVID-19 vaccine before or after COVID-19 infection (heterogeneity *P* = .38, I^2^ = 5%), and homogenous for studies evaluating post-COVID conditions in individuals receiving vaccine only before infection (heterogeneity *P* = .26, I^2^ = 25%), and also homogenous for studies evaluating post-COVID conditions in children who received 2 doses (heterogeneity *P* = .77, I^2^ = 0%), respectively. There was no evidence for publication bias among the 5 studies included in the meta-analysis (*P* = .93).

**Table 2.** Subset analyses evaluating COVID-19 vaccine effectiveness against post-COVID conditions in individuals who received COVID-19 vaccine before or after COVID-19 infection

# of COVID-19 vaccine doses	COVID-19 vaccine before/after COVID-19 infection	Studies included (n)	Participants (n)	Pooled diagnostic odds ratio (DOR) (95% CI)	I^2^ test for heterogeneity
At least 1 dose	Before/after	5^11^,^12^,^20^,^23^,^24^	20,325	1.073 (0.772–1.492)	5%
At least 1 dose	Before	3^12^,^20^,^24^	12,095	1.225 (0.835–1.799)	25%
Two doses	Before	2^12^,^24^	2,036	0.820 (0.625–1.075)	0%

Note. COVID-19, coronavirus disease 2019; CI, confidence interval.

One dose – before /after COVID-19 infection: 5 studies (Atchison 2023, Lokanuwatsatien 2023, Messiah 2022, Morello 2023, Pinto Pereira 2023).

One dose – before COVID-19 infection: 3 studies (Atchison 2023, Lokanuwatsatien 2023, Morello 2023).

Two doses – before COVID-19 infection: 2 studies (Lokanuwatsatien 2023, Morello 2023).

Regarding the quality assessment scores of the 8 included studies, the great majority of the studies (6 studies) were considered good quality (19–23 of 28 possible points)^12,19–21,23,24^ per the Downs and Black quality tool, and 2 studies were considered fair (14–18 points).^11,22^

## Discussion

Our systematic literature review and meta-analysis aimed to assess the VE of COVID-19 vaccination in mitigating post-COVID conditions in children and adolescents. The prevalence of those who did not receive COVID-19 vaccines was high, ranging from 65% to 97%. The pooled prevalence of post-COVID conditions was similar between unvaccinated (21.3%) and those who received at least 1 dose (20.3%).^10,11,19,22,23^ This meta-analysis did not demonstrate statistically significant protection with COVID-19 vaccination against post-COVID conditions in the pediatric population. Given the limited number of studies included in this meta-analysis, each with different criteria for defining post-COVID conditions, further research is necessary to comprehensively assess VE.

The pursuit of effective vaccination strategies against COVID-19, particularly for children and adolescents, has been a crucial aspect of the global response to the pandemic.^1,7,25^ The low prevalence of COVID-19 vaccination among the pediatric population is a concerning issue that warrants further examination.^11,12,20,23,24^ Initially, COVID-19 vaccines were primarily authorized for adults, and the distribution to children and adolescents occurred later.^25^ Certainly, understanding parental hesitancy regarding COVID-19 vaccination for their children and adolescents is a critical aspect to consider in the context of this study.^26^ Additionally, acknowledging that the pediatric population was one of the last to receive COVID-19 vaccines adds complexity to the discussion. The delayed access of children and adolescents to COVID-19 vaccines might have contributed to a sense of uncertainty among parents. They may have been waiting for more data and real-world evidence of vaccine safety and efficacy in this age group before making decisions.^27^ The spread of misinformation and disinformation about COVID-19 vaccines has contributed to vaccine hesitancy in both adults and children.^26^ False claims about vaccine safety, efficacy, and adverse events can lead parents to make uninformed decisions about vaccinating their children.^26^ Highlighting the potential risks of post-COVID conditions in adult populations can be a persuasive argument for vaccination.^28^ Parents may be more inclined to vaccinate their children if they understand the potential long-term health consequences of a COVID-19 infection, even if it is mild.^4,28^ Moreover, the study’s focus on pediatric populations highlights the need for age-specific considerations in vaccination campaigns.^7,25,29^

The observed wide variation in the prevalence of post-COVID conditions in pediatric populations, ranging from 1.6% to 70%, underscores the complexity of this issue,^4^ possibly influenced by an individual’s age, health conditions, and viral variants problems.^28,30^ Our previous systematic review and meta-analysis evaluating the VE against post-COVID conditions among fully vaccinated adults revealed that the pooled prevalence of post-COVID conditions was 11.8% among those who were unvaccinated and 5.3% among those individuals who were fully vaccinated.^8^ This present systematic review demonstrated that the pediatric population had a higher prevalence at 21% in unvaccinated and 20% in vaccinated pediatric patients. The higher prevalence of post-COVID conditions in pediatric populations compared with adults is a matter of concern and requires careful consideration. Several factors could contribute to this difference.^25,30,31^ Emerging variants could potentially have different effects on post-COVID conditions, and their prevalence may influence VE.^32^ Prior research indicated that the Delta and Omicron variants led to fewer systemic inflammatory responses, severe illnesses, or fatalities, thereby resulting in milder long COVID symptoms compared with the original wild-type variant (Wuhan).^33,34^ In addition, it has been reported that the prevalence of post-COVID conditions was lower during the Omicron era than during other strains, and the prior systematic review among adults might have included more research that covered the additional newer variants.^34,35^ The variability in how post-COVID conditions were defined across the included studies poses a significant limitation for understanding this issue. Different studies used different time thresholds (4 weeks, 12 weeks, 6 months) to define post-COVID conditions.^11,12,19–24^ This heterogeneity in definitions makes it challenging to compare results across studies and draw firm conclusions about the prevalence and impact of these conditions.

While our previous meta-analysis among the adult population suggested that COVID-19 vaccines might effectively prevent post-COVID-19 conditions,^36^ the present meta-analysis did not demonstrate any protective effect. There are a few potential reasons for this discrepancy. Children and adolescents have unique physiological and immunological characteristics, which may influence vaccine responses and the risk of post-COVID conditions.^25^ It is worth noting that there have been previous studies that have also shown limited effectiveness of vaccines in preventing post-COVID conditions.^11,12,20,24^ These findings suggest that the relationship between vaccination and post-COVID outcomes is complex and may vary across different age groups and populations. Moreover, the timing of vaccination concerning the COVID-19 infection may be a contributing factor. Some studies have analyzed VE when administered before COVID-19 infection,^12,20^ while others have assessed outcomes when given after infection.^11,22^ These differences in timing may contribute to the divergent results observed in different studies. Consequently, ongoing research and monitoring of vaccine safety and effectiveness in these age groups are essential for refining vaccination strategies.

Our study had several limitations. First, there were a relatively small number of articles that met the inclusion criteria. With only 8 studies included in the systematic literature review,^11,12,19–24^ the available evidence may not represent the full spectrum of the pediatric population’s experience with post-COVID conditions. All of the included studies in the meta-analysis investigating the VE in preventing post-COVID conditions employ non-randomized and different observational study designs,^11,12,20,23,24^ including cohort studies^11,12,23,24^ and a cross-sectional study.^20^ While the authors applied rigorous quality assessment criteria, the possibility of residual confounding factors and selection of bias that were not accounted for in the original studies remains. Additionally, it’s important to consider that children and adolescents who were sicker, frailer, or at higher risk might have been more inclined to receive vaccinations. This potential bias is difficult to completely account for in observational studies. Second, the articles included in this systematic literature review assessed the effectiveness of various COVID-19 vaccines, including mRNA vaccines, vector vaccines, and inactivated viral vaccines. The effectiveness of these vaccines in preventing post-COVID conditions may differ, but the analysis did not stratify results by vaccine type due to data limitations. Future research should explore potential differences in the impact of different vaccine types on post-COVID outcomes. Third, the studies included in our systematic literature review were conducted in various countries, with differing healthcare systems, demographics, COVID-19 vaccination programs, and COVID-19 prevalence. The diversity in settings could affect the generalizability of the findings to other regions or populations. It is important to consider that the impact of vaccines on post-COVID conditions may vary in different contexts.^30,36^ Lastly, although no evidence of publication bias was found in the meta-analysis, the potential for publication bias always exists in systematic reviews. Studies with significant findings, whether positive or negative, may be more likely to be published, potentially skewing the overall assessment of VE.

In conclusion, it appears that the pediatric population may experience a higher incidence of post-COVID conditions compared with adults. The study observed that COVID-19 vaccination, whether administered before or after a COVID-19 infection, did not reduce the occurrence of post-COVID conditions associated with the circulating variants during the study period among pediatric individuals. While this article contributes to our understanding of COVID-19 VE in preventing post-COVID conditions in the pediatric population, it is crucial to acknowledge the limitations of this study. Addressing these limitations through more extensive, standardized, and regionally diverse research, along with ongoing monitoring and robust study designs, will offer a more accurate insight into the relationship between vaccination and post-COVID outcomes in children and adolescents. Such efforts are vital for refining public health strategies and mitigating the long-term health effects of the pandemic among younger populations.

## Supporting information

Gutfreund et al. supplementary material 1 Gutfreund et al. supplementary material

Gutfreund et al. supplementary material 2 Gutfreund et al. supplementary material

Gutfreund et al. supplementary material 3 Gutfreund et al. supplementary material

Gutfreund et al. supplementary material 4 Gutfreund et al. supplementary material

## Supplementary material

The supplementary material for this article can be found at https://doi.org/10.1017/ash.2024.42.
