# Supplementary material for: The effectiveness of the COVID-19 vaccines in the prevention of post-COVID conditions in children and adolescents: a systematic literature review and meta-analysis
Source: Antimicrob Steward Healthc Epidemiol. 2024 Apr 19;4(1):e54. doi: 10.1017/ash.2024.42 (PMC11036435; doi:10.1017/ash.2024.42)
Supplement: Gutfreund et al. supplementary material 1 — Gutfreund et al. supplementary material [file S2732494X24000421sup001.docx]

**Supplementary Appendix 1**. Search strategy
