# Supplementary material for: The effectiveness of the COVID-19 vaccines in the prevention of post-COVID conditions in children and adolescents: a systematic literature review and meta-analysis
Source: Antimicrob Steward Healthc Epidemiol. 2024 Apr 19;4(1):e54. doi: 10.1017/ash.2024.42 (PMC11036435; doi:10.1017/ash.2024.42)
Supplement: Gutfreund et al. supplementary material 4 — Gutfreund et al. supplementary material [file S2732494X24000421sup004.docx]

**PubMed 8/14/23**

#1

(COVID [Title/Abstract] OR Covid 19 [Title/Abstract] OR Corona virus [Title/Abstract] OR Coronavirus [Title/Abstract] OR 2019-nCoV [Title/Abstract] OR SARS-CoV-2 [Title/Abstract] OR 2019-nCoV[Title/Abstract]) AND (long duration [Title/Abstract] OR persistent symptoms [Title/Abstract] OR post acute sequelae [Title/Abstract] OR postacute sequelae[Title/Abstract])

OR

"Post-Acute COVID-19 Syndrome"[Mesh] OR "COVID-19/complications"[Mesh] OR PASC [Title/Abstract] OR Post acute COVID [Title/Abstract] OR Postacute COVID[Title/Abstract] OR "long covid"[Title/Abstract:~4] OR "chronic covid" [Title/Abstract:~4] OR "persistent covid" [Title/Abstract:~4] OR "covid complications"[Title/Abstract:~4] OR "post covid"[Title/Abstract:~4]

#2

"Vaccine Efficacy"[Mesh] OR "Disease Progression"[Mesh] OR "Hospitalization"[Mesh] OR protect*[Title/Abstract] OR prevent*[Title/Abstract] OR associat*[Title/Abstract] OR correlat*[Title/Abstract] OR efficacy[Title/Abstract] OR effect*[Title/Abstract] OR improve*[Title/Abstract] OR immunogenicity[tw] OR antibod*[tw] OR symptom*[tw] OR health[tw]

#3

"Immunization"[Mesh] OR "COVID-19 Vaccines"[Mesh] OR vaccin*[Title/Abstract] OR unvaccinated[Title/Abstract] OR immunization*[Title/Abstract] OR variolation*[Title/Abstract] OR "Immunologic stimulation"[Title/Abstract] OR Immunostimulation[Title/Abstract]

#4

Infant[Mesh:NoExp] OR Infant*[tw] OR infancy[tw] OR Baby[tw] OR Babies[tw] OR Child[MeSH] OR Child[tw] OR children[tw] OR Toddler*[tw] OR Adolescent[MeSH] OR Adoles*[tw] OR Teen*[tw] OR Boy[tw] OR boys[tw] OR Girl*[tw] OR Pediatrics[MeSH] OR Pediatric*[tw] OR Paediatric*[tw]

#1 AND #2 AND #3 AND #4=518 with date filter 2019-present

**CINAHL 8/14/23**

#1

(MH "Post-Acute COVID-19 Syndrome") OR (MH "COVID-19+/CO") OR TI ( "Post acute sequelae" OR "Post acute COVID" OR PASC OR "Postacute COVID") OR AB ( "Post acute sequelae" OR "Post acute COVID" OR PASC OR "Postacute COVID") OR (chronic OR long OR expanded OR extended OR recurr* OR sustain* OR persist* OR prolong* OR continu* OR debilitating) N3 (covid OR coronavirus OR "corona virus" OR Sars Cov 2 OR 2019 nCOV)

#2

TI ( Vaccinat* OR Unvaccinated OR vaccine* OR immunization* OR variolation* OR "Immunologic stimulation" OR Immunostimulation) OR AB (Vaccinat* OR Unvaccinated OR vaccine* OR immunization* OR variolation* OR "Immunologic stimulation" OR Immunostimulation)

OR

MH "Immunization+" OR MH "COVID-19 Vaccines"

#3

MH "Disease Progression+" OR MH "Vaccine Efficacy" OR MH "Hospitalization+" OR protect* OR prevent* OR associat* OR correlat* OR efficacy OR effect* OR improve* OR immunogenicity OR antibod* OR symptom* OR health

#4

MH "Child+" OR MH "Adolescence+" OR Infant* OR infancy OR Baby OR Babies OR Child[MeSH] OR Child OR children OR Toddler* OR Adoles* OR Teen* OR Boy OR boys OR Girl* OR Pediatrics[MeSH] OR Pediatric* OR Paediatric*

#1 AND #2 AND #3 AND #4=148 pub date 2019 to present

**Embase 8/14/23**

#1

'coronavirus disease 2019'/exp/dm_co OR 'long covid'/exp OR 'post acute sequelae':ab,ti OR 'post acute covid':ab,ti OR pasc:ab,ti OR 'postacute covid':ab,ti OR (long OR chronic OR persistent OR recurrent OR prolonged) NEAR/2 (covid OR coronavirus OR 'corona virus' OR 'sars cov 2' OR '2019 ncov')

#2

'immunization'/exp OR 'sars-cov-2 vaccine'/exp OR vaccinat*:ti,ab OR unvaccinated:ti,ab OR vaccine*:ti,ab OR immunization*:ti,ab OR variolation*:ti,ab OR 'immunologic stimulation':ti,ab OR immunostimulation:ti,ab

#3

'hospitalization'/exp OR 'disease exacerbation'/exp OR 'risk factor'/exp OR protect*:ab,ti OR prevent*:ab,ti OR associat*:ab,ti OR correlat*:ab,ti OR efficacy:ab,ti OR effect*:ab,ti OR improve*:ab,ti OR health:ab,ti OR antibod*:ab,ti OR immunogenicity:ab,ti OR symptom*:ab,ti

#4

'child'/exp OR 'adolescent'/exp OR 'pediatrics'/exp OR infant* OR infancy OR baby OR babies OR child OR children OR toddler* OR adoles* OR teen* OR pediatric* OR paediatric* OR boy* OR girl*

#1 AND #2 AND #3 AND #4 with pub dates 2019-2023= 337

**Web of Science**

TS=(vaccine* OR vaccination*OR pfizer OR moderna OR immunization* OR  variolation* OR  "immunologic stimulation"OR immunostimulation  OR  gamaleya  OR  "Sputnik V" OR sinovac OR "Corona Vac" OR astrazeneca OR janssen OR "AZD1222"  OR "mRNA-1273" OR janssen OR "Johnson & Johnson" OR "JNJ-78436735")

#2

TS=(((long NEAR/3 covid) OR (chronic NEAR/3 covid) OR (recurrent NEAR/3 covid) OR (persistent NEAR/3 covid) OR (postacute NEAR/3 covid) OR (post acute NEAR/3 covid) OR PASC OR post acute sequelae))

#3

TS= (infant* OR infancy OR baby OR babies OR child OR children OR toddler* OR adoles* OR teen* OR pediatric* OR paediatric* OR boy* OR girl*)

#1 AND #2 AND #3=91

**Cochrane CENTRAL 8/14/23**

**#**1 MeSH descriptor: [Post-Acute COVID-19 Syndrome] explode all trees

#2 MeSH descriptor: [COVID-19] explode all trees and with qualifier(s): [complications - CO]

#3 (post OR postacute OR long OR recurrent OR prolonged OR post acute OR PASC):ti,ab,kw AND (covid OR coronavirus):ti,ab,kw (Word variations have been searched)

#4 MeSH descriptor: [COVID-19 Vaccines] explode all trees

#5 MeSH descriptor: [Vaccine Efficacy] explode all trees

#6 MeSH descriptor: [Adolescent] explode all trees

#7 MeSH descriptor: [Child] explode all trees

#8 (vaccine* OR vaccination* OR immunization* OR variolation* OR "immunologic stimulation" OR immunostimulation):ti,ab,kw (Word variations have been searched)

#9 (infant* OR infancy OR baby OR babies OR child OR children OR toddler* OR adoles* OR teen* OR pediatric* OR paediatric* OR boy* OR girl*):ti,ab,kw (Word variations have been searched)

#10 #1 OR #2 OR #3= 4710

#11 #4 OR #5 OR #8= 33077

#12 #6 OR #7 OR #9= 330390

#13 #10 AND #11 AND #12=119, 116 limited to trials

**Scopus 8/14/23**

citing paper search for known inclusions=29
